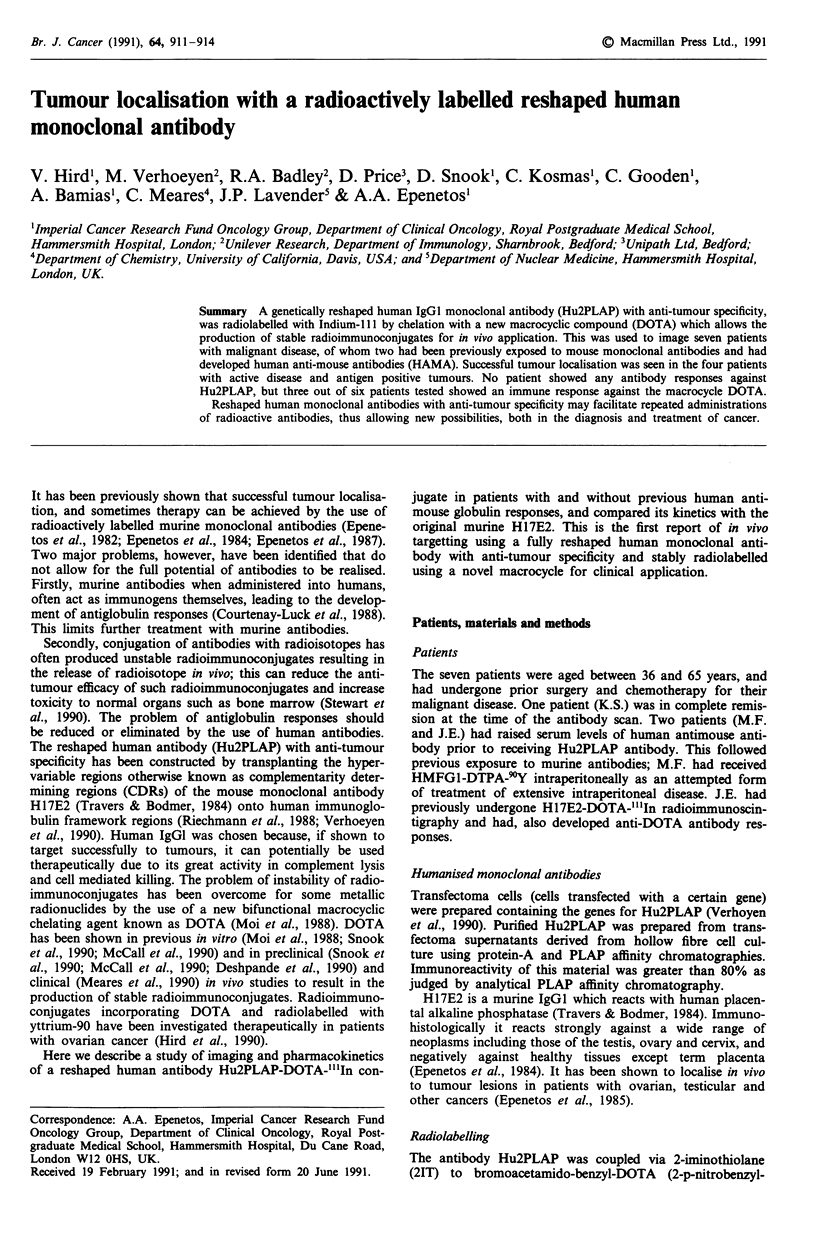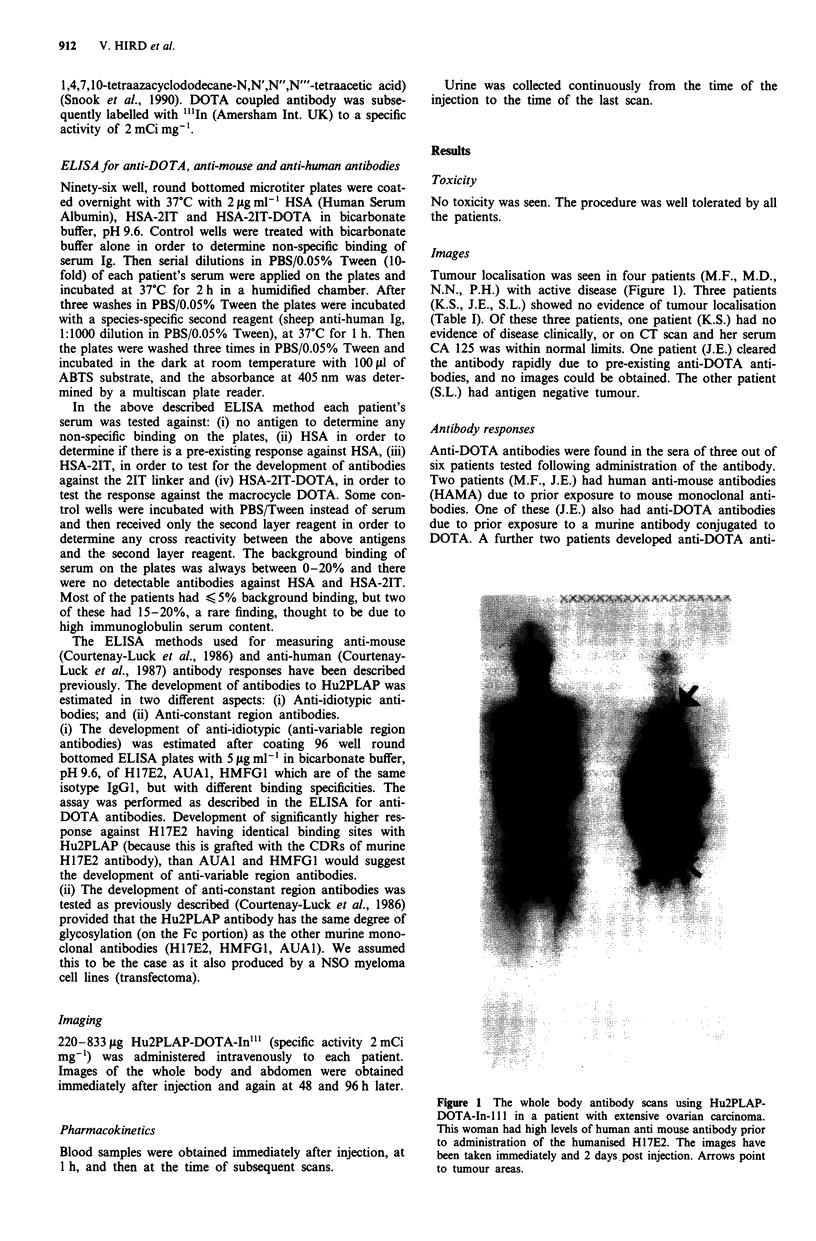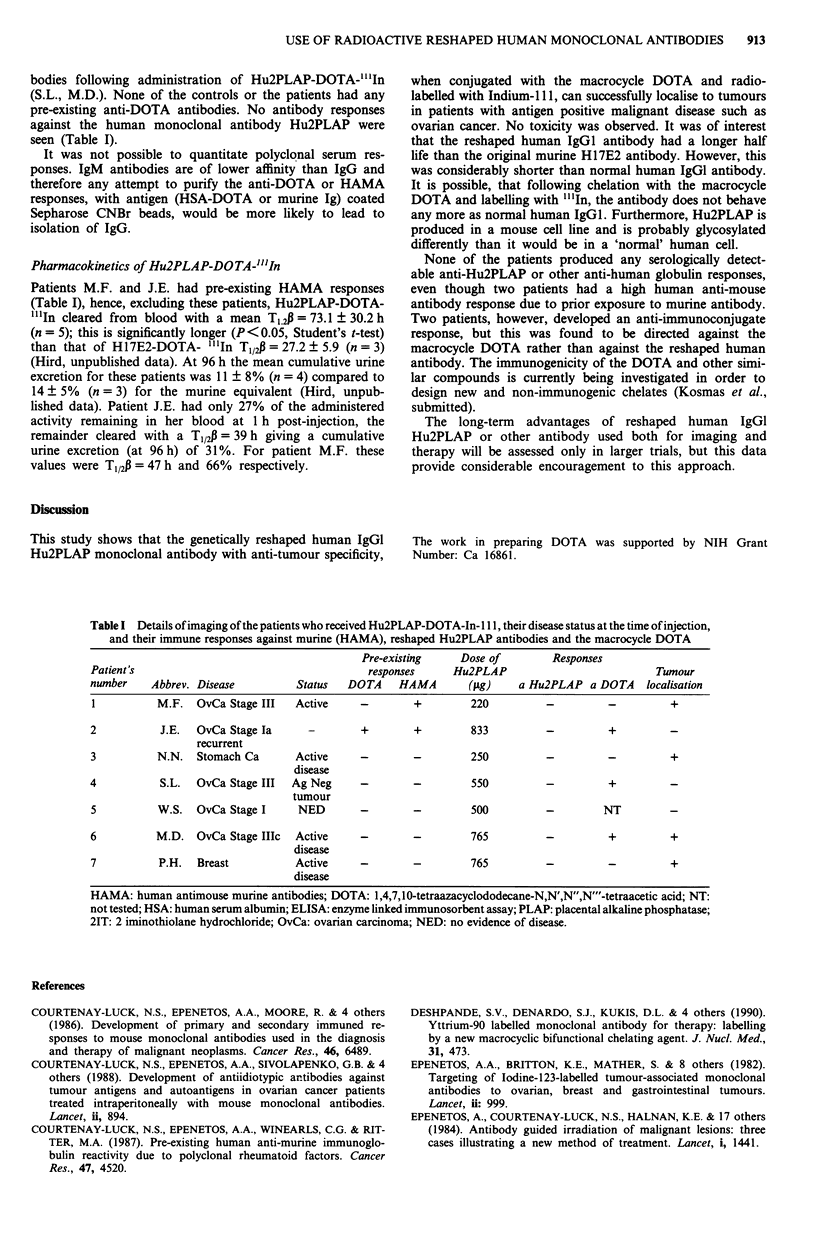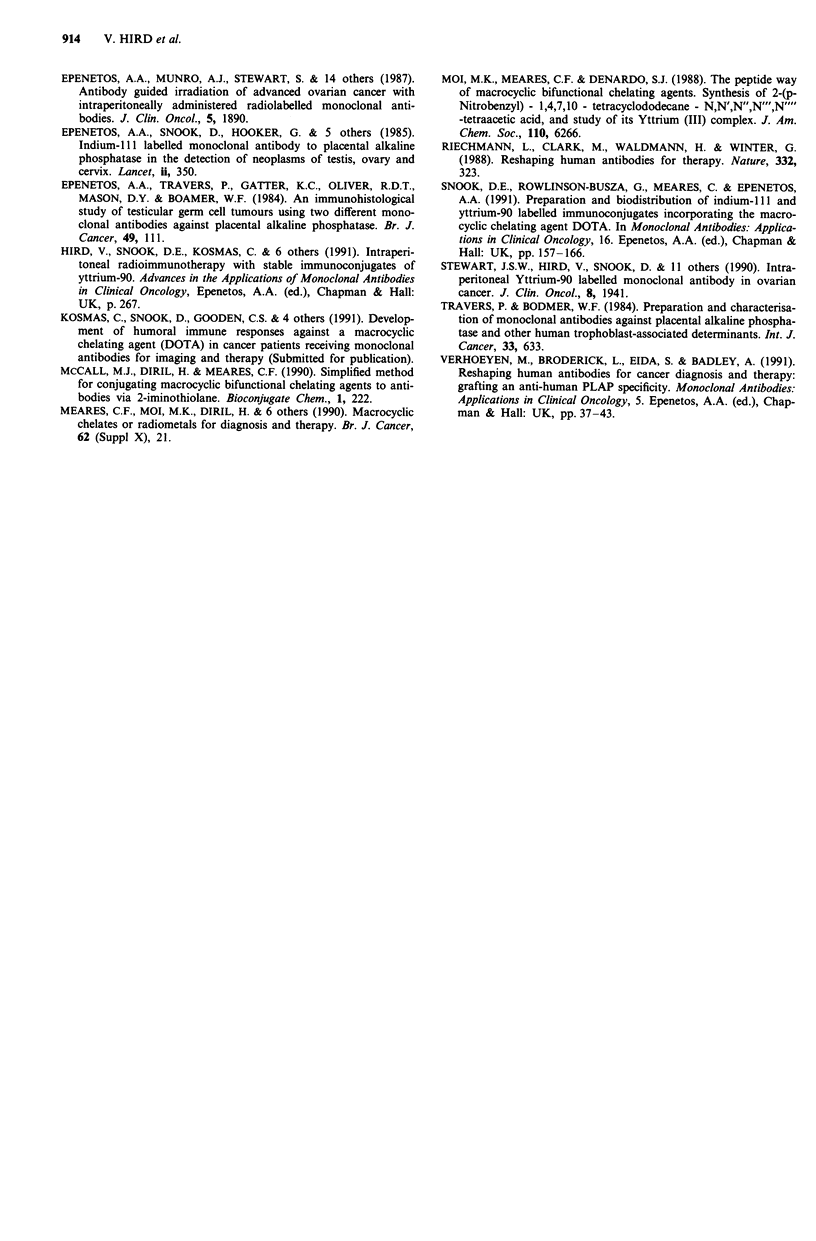# Tumour localisation with a radioactively labelled reshaped human monoclonal antibody.

**DOI:** 10.1038/bjc.1991.425

**Published:** 1991-11

**Authors:** V. Hird, M. Verhoeyen, R. A. Badley, D. Price, D. Snook, C. Kosmas, C. Gooden, A. Bamias, C. Meares, J. P. Lavender

**Affiliations:** Imperial Cancer Research Fund Oncology Group, Department of Clinical Oncology, Royal Postgraduate Medical School, Hammersmith Hospital, London, UK.

## Abstract

**Images:**


Br. J. Cancer (1991), 64, 911-914 © Macmillan Press Ltd., 1991

### Tumour localisation with a radioactively labelled reshaped human monoclonal antibody

V. Hird[1], M. Verhoeyen[2], R.A. Badley[2], D. Price[3], D. Snook[1], C. Kosmas[1], C. Gooden[1], A. Bamias[1], C. Meares[4], J.P. Lavender[5] & A.A. Epenetos[1]

[1]*Imperial Cancer Research Fund Oncology Group, Department of Clinical Oncology, Royal Postgraduate Medical School, Hammersmith Hospital, London;* [2]*Unilever Research, Department of Immunology, Sharnbrook, Bedford;* [3]*Unipath Ltd, Bedford;* [4]*Department of Chemistry, University of California, Davis, USA; and* [5]*Department of Nuclear Medicine, Hammersmith Hospital, London, UK.*

**Summary** A genetically reshaped human IgG1 monoclonal antibody (Hu2PLAP) with anti-tumour specificity, was radiolabelled with Indium-111 by chelation with a new macrocyclic compound (DOTA) which allows the production of stable radioimmunoconjugates for *in vivo* application. This was used to image seven patients with malignant disease, of whom two had been previously exposed to mouse monoclonal antibodies and had developed human anti-mouse antibodies (HAMA). Successful tumour localisation was seen in the four patients with active disease and antigen positive tumours. No patient showed any antibody responses against Hu2PLAP, but three out of six patients tested showed an immune response against the macrocycle DOTA. Reshaped human monoclonal antibodies with anti-tumour specificity may facilitate repeated administrations of radioactive antibodies, thus allowing new possibilities, both in the diagnosis and treatment of cancer.

It has been previously shown that successful tumour localisation, and sometimes therapy can be achieved by the use of radioactively labelled murine monoclonal antibodies (Epenetos *et al.*, 1982; Epenetos *et al.*, 1984; Epenetos *et al.*, 1987). Two major problems, however, have been identified that do not allow for the full potential of antibodies to be realised. Firstly, murine antibodies when administered into humans, often act as immunogens themselves, leading to the development of antiglobulin responses (Courtenay-Luck *et al.*, 1988). This limits further treatment with murine antibodies.

Secondly, conjugation of antibodies with radioisotopes has often produced unstable radioimmunoconjugates resulting in the release of radioisotope *in vivo*; this can reduce the anti-tumour efficacy of such radioimmunoconjugates and increase toxicity to normal organs such as bone marrow (Stewart *et al.*, 1990). The problem of antiglobulin responses should be reduced or eliminated by the use of human antibodies. The reshaped human antibody (Hu2PLAP) with anti-tumour specificity has been constructed by transplanting the hypervariable regions otherwise known as complementarity determining regions (CDRs) of the mouse monoclonal antibody H17E2 (Travers & Bodmer, 1984) onto human immunoglobulin framework regions (Riechmann *et al.*, 1988; Verhoeyen *et al.*, 1990). Human IgGl was chosen because, if shown to target successfully to tumours, it can potentially be used therapeutically due to its great activity in complement lysis and cell mediated killing. The problem of instability of radioimmunoconjugates has been overcome for some metallic radionuclides by the use of a new bifunctional macrocyclic chelating agent known as DOTA (Moi *et al.*, 1988). DOTA has been shown in previous *in vitro* (Moi *et al.*, 1988; Snook *et al.*, 1990; McCall *et al.*, 1990) and in preclinical (Snook *et al.*, 1990; McCall *et al.*, 1990; Deshpande *et al.*, 1990) and clinical (Meares *et al.*, 1990) *in vivo* studies to result in the production of stable radioimmunoconjugates. Radioimmunoconjugates incorporating DOTA and radiolabelled with yttrium-90 have been investigated therapeutically in patients with ovarian cancer (Hird *et al.*, 1990).

Here we describe a study of imaging and pharmacokinetics of a reshaped human antibody Hu2PLAP-DOTA-$^{111}$In conjugate in patients with and without previous human anti-mouse globulin responses, and compared its kinetics with the original murine H17E2. This is the first report of *in vivo* targetting using a fully reshaped human monoclonal antibody with anti-tumour specificity and stably radiolabelled using a novel macrocycle for clinical application.

Correspondence: A.A. Epenetos, Imperial Cancer Research Fund Oncology Group, Department of Clinical Oncology, Royal Postgraduate Medical School, Hammersmith Hospital, Du Cane Road, London W12 0HS, UK.
Received 19 February 1991; and in revised form 20 June 1991.

### Patients, materials and methods

### *Patients*

The seven patients were aged between 36 and 65 years, and had undergone prior surgery and chemotherapy for their malignant disease. One patient (K.S.) was in complete remission at the time of the antibody scan. Two patients (M.F. and J.E.) had raised serum levels of human antimouse antibody prior to receiving Hu2PLAP antibody. This followed previous exposure to murine antibodies; M.F. had received HMFG1-DTPA-$^{90}$Y intraperitoneally as an attempted form of treatment of extensive intraperitoneal disease. J.E. had previously undergone H17E2-DOTA-$^{111}$In radioimmunoscintigraphy and had, also developed anti-DOTA antibody responses.

### *Humanised monoclonal antibodies*

Transfectoma cells (cells transfected with a certain gene) were prepared containing the genes for Hu2PLAP (Verhoyen *et al.*, 1990). Purified Hu2PLAP was prepared from transfectoma supernatants derived from hollow fibre cell culture using protein-A and PLAP affinity chromatographies. Immunoreactivity of this material was greater than 80% as judged by analytical PLAP affinity chromatography.

H17E2 is a murine IgG1 which reacts with human placental alkaline phosphatase (Travers & Bodmer, 1984). Immunohistologically it reacts strongly against a wide range of neoplasms including those of the testis, ovary and cervix, and negatively against healthy tissues except term placenta (Epenetos *et al.*, 1984). It has been shown to localise *in vivo* to tumour lesions in patients with ovarian, testicular and other cancers (Epenetos *et al.*, 1985).

### *Radiolabelling*

The antibody Hu2PLAP was coupled via 2-iminothiolane (2IT) to bromoacetamido-benzyl-DOTA (2-p-nitrobenzyl-

1,4,7,10-tetraazacyclododecane-N,N',N'',N'''-tetraacetic acid) (Snook et al., 1990). DOTA coupled antibody was subsequently labelled with $^{111}$In (Amersham Int. UK) to a specific activity of 2 mCi $mg^{-1}$.

*ELISA for anti-DOTA, anti-mouse and anti-human antibodies*

Ninety-six well, round bottomed microtiter plates were coated overnight with 37°C with 2 $\mu g\,ml^{-1}$ HSA (Human Serum Albumin), HSA-2IT and HSA-2IT-DOTA in bicarbonate buffer, pH 9.6. Control wells were treated with bicarbonate buffer alone in order to determine non-specific binding of serum Ig. Then serial dilutions in PBS/0.05% Tween (10-fold) of each patient's serum were applied on the plates and incubated at 37°C for 2 h in a humidified chamber. After three washes in PBS/0.05% Tween the plates were incubated with a species-specific second reagent (sheep anti-human Ig, 1:1000 dilution in PBS/0.05% Tween), at 37°C for 1 h. Then the plates were washed three times in PBS/0.05% Tween and incubated in the dark at room temperature with 100 µl of ABTS substrate, and the absorbance at 405 nm was determined by a multiscan plate reader.

In the above described ELISA method each patient's serum was tested against: (i) no antigen to determine any non-specific binding on the plates, (ii) HSA in order to determine if there is a pre-existing response against HSA, (iii) HSA-2IT, in order to test for the development of antibodies against the 2IT linker and (iv) HSA-2IT-DOTA, in order to test the response against the macrocycle DOTA. Some control wells were incubated with PBS/Tween instead of serum and then received only the second layer reagent in order to determine any cross reactivity between the above antigens and the second layer reagent. The background binding of serum on the plates was always between 0–20% and there were no detectable antibodies against HSA and HSA-2IT. Most of the patients had $\leqslant 5\%$ background binding, but two of these had 15–20%, a rare finding, thought to be due to high immunoglobulin serum content.

The ELISA methods used for measuring anti-mouse (Courtenay-Luck et al., 1986) and anti-human (Courtenay-Luck et al., 1987) antibody responses have been described previously. The development of antibodies to Hu2PLAP was estimated in two different aspects: (i) Anti-idiotypic antibodies; and (ii) Anti-constant region antibodies.

(i) The development of anti-idiotypic (anti-variable region antibodies) was estimated after coating 96 well round bottomed ELISA plates with 5 $\mu g\,ml^{-1}$ in bicarbonate buffer, pH 9.6, of H17E2, AUA1, HMFG1 which are of the same isotype IgG1, but with different binding specificities. The assay was performed as described in the ELISA for anti-DOTA antibodies. Development of significantly higher response against H17E2 having identical binding sites with Hu2PLAP (because this is grafted with the CDRs of murine H17E2 antibody), than AUA1 and HMFG1 would suggest the development of anti-variable region antibodies.

(ii) The development of anti-constant region antibodies was tested as previously described (Courtenay-Luck et al., 1986) provided that the Hu2PLAP antibody has the same degree of glycosylation (on the Fc portion) as the other murine monoclonal antibodies (H17E2, HMFG1, AUA1). We assumed this to be the case as it also produced by a NSO myeloma cell lines (transfectoma).

*Imaging*

220–833 µg Hu2PLAP-DOTA-In$^{111}$ (specific activity 2 mCi $mg^{-1}$) was administered intravenously to each patient. Images of the whole body and abdomen were obtained immediately after injection and again at 48 and 96 h later.

*Pharmacokinetics*

Blood samples were obtained immediately after injection, at 1 h, and then at the time of subsequent scans.

Urine was collected continuously from the time of the injection to the time of the last scan.

### Results

*Toxicity*

No toxicity was seen. The procedure was well tolerated by all the patients.

*Images*

Tumour localisation was seen in four patients (M.F., M.D., N.N., P.H.) with active disease (Figure 1). Three patients (K.S., J.E., S.L.) showed no evidence of tumour localisation (Table I). Of these three patients, one patient (K.S.) had no evidence of disease clinically, or on CT scan and her serum CA 125 was within normal limits. One patient (J.E.) cleared the antibody rapidly due to pre-existing anti-DOTA antibodies, and no images could be obtained. The other patient (S.L.) had antigen negative tumour.

*Antibody responses*

Anti-DOTA antibodies were found in the sera of three out of six patients tested following administration of the antibody. Two patients (M.F., J.E.) had human anti-mouse antibodies (HAMA) due to prior exposure to mouse monoclonal antibodies. One of these (J.E.) also had anti-DOTA antibodies due to prior exposure to a murine antibody conjugated to DOTA. A further two patients developed anti-DOTA anti-

**Figure 1** The whole body antibody scans using Hu2PLAP-DOTA-In-111 in a patient with extensive ovarian carcinoma. This woman had high levels of human anti mouse antibody prior to administration of the humanised H17E2. The images have been taken immediately and 2 days post injection. Arrows point to tumour areas.

bodies following administration of Hu2PLAP-DOTA-$^{111}$In (S.L., M.D.). None of the controls or the patients had any pre-existing anti-DOTA antibodies. No antibody responses against the human monoclonal antibody Hu2PLAP were seen (Table I).

It was not possible to quantitate polyclonal serum responses. IgM antibodies are of lower affinity than IgG and therefore any attempt to purify the anti-DOTA or HAMA responses, with antigen (HSA-DOTA or murine Ig) coated Sepharose CNBr beads, would be more likely to lead to isolation of IgG.

*Pharmacokinetics of Hu2PLAP-DOTA-$^{111}$In*

Patients M.F. and J.E. had pre-existing HAMA responses (Table I), hence, excluding these patients, Hu2PLAP-DOTA-$^{111}$In cleared from blood with a mean $T_{1/2}\beta = 73.1 \pm 30.2$ h ($n = 5$); this is significantly longer ($P < 0.05$, Student's $t$-test) than that of H17E2-DOTA- $^{111}$In $T_{1/2}\beta = 27.2 \pm 5.9$ ($n = 3$) (Hird, unpublished data). At 96 h the mean cumulative urine excretion for these patients was $11 \pm 8\%$ ($n = 4$) compared to $14 \pm 5\%$ ($n = 3$) for the murine equivalent (Hird, unpublished data). Patient J.E. had only 27% of the administered activity remaining in her blood at 1 h post-injection, the remainder cleared with a $T_{1/2}\beta = 39$ h giving a cumulative urine excretion (at 96 h) of 31%. For patient M.F. these values were $T_{1/2}\beta = 47$ h and 66% respectively.

### Discussion

This study shows that the genetically reshaped human IgGl Hu2PLAP monoclonal antibody with anti-tumour specificity, when conjugated with the macrocycle DOTA and radiolabelled with Indium-111, can successfully localise to tumours in patients with antigen positive malignant disease such as ovarian cancer. No toxicity was observed. It was of interest that the reshaped human IgG1 antibody had a longer half life than the original murine H17E2 antibody. However, this was considerably shorter than normal human IgGl antibody. It is possible, that following chelation with the macrocycle DOTA and labelling with $^{111}$In, the antibody does not behave any more as normal human IgG1. Furthermore, Hu2PLAP is produced in a mouse cell line and is probably glycosylated differently than it would be in a 'normal' human cell.

None of the patients produced any serologically detectable anti-Hu2PLAP or other anti-human globulin responses, even though two patients had a high human anti-mouse antibody response due to prior exposure to murine antibody. Two patients, however, developed an anti-immunoconjugate response, but this was found to be directed against the macrocycle DOTA rather than against the reshaped human antibody. The immunogenicity of the DOTA and other similar compounds is currently being investigated in order to design new and non-immunogenic chelates (Kosmas *et al.*, submitted).

The long-term advantages of reshaped human IgGl Hu2PLAP or other antibody used both for imaging and therapy will be assessed only in larger trials, but this data provide considerable encouragement to this approach.

The work in preparing DOTA was supported by NIH Grant Number: Ca 16861.

**Table I** Details of imaging of the patients who received Hu2PLAP-DOTA-In-111, their disease status at the time of injection, and their immune responses against murine (HAMA), reshaped Hu2PLAP antibodies and the macrocycle DOTA

| *Patient's number* | *Abbrev.* | *Disease* | *Status* | *Pre-existing responses* DOTA | *Pre-existing responses* HAMA | *Dose of Hu2PLAP* (μg) | *Responses* a Hu2PLAP | *Responses* a DOTA | *Tumour localisation* |
|---|---|---|---|---|---|---|---|---|---|
| 1 | M.F. | OvCa Stage III | Active | – | + | 220 | – | – | + |
| 2 | J.E. | OvCa Stage Ia recurrent | - | + | + | 833 | – | + | – |
| 3 | N.N. | Stomach Ca | Active disease | – | – | 250 | – | – | + |
| 4 | S.L. | OvCa Stage III | Ag Neg tumour | – | – | 550 | – | + | – |
| 5 | W.S. | OvCa Stage I | NED | – | – | 500 | – | NT | – |
| 6 | M.D. | OvCa Stage IIIc | Active disease | – | – | 765 | – | + | + |
| 7 | P.H. | Breast | Active disease | – | – | 765 | – | – | + |

HAMA: human antimouse murine antibodies; DOTA: 1,4,7,10-tetraazacyclododecane-N,N',N'',N'''-tetraacetic acid; NT: not tested; HSA: human serum albumin; ELISA: enzyme linked immunosorbent assay; PLAP: placental alkaline phosphatase; 2IT: 2 iminothiolane hydrochloride; OvCa: ovarian carcinoma; NED: no evidence of disease.

### References

COURTENAY-LUCK, N.S., EPENETOS, A.A., MOORE, R. & 4 others (1986). Development of primary and secondary immuned responses to mouse monoclonal antibodies used in the diagnosis and therapy of malignant neoplasms. *Cancer Res.*, **46**, 6489.

COURTENAY-LUCK, N.S., EPENETOS, A.A., SIVOLAPENKO, G.B. & 4 others (1988). Development of antiidiotypic antibodies against tumour antigens and autoantigens in ovarian cancer patients treated intraperitoneally with mouse monoclonal antibodies. *Lancet*, **ii**, 894.

COURTENAY-LUCK, N.S., EPENETOS, A.A., WINEARLS, C.G. & RITTER, M.A. (1987). Pre-existing human anti-murine immunoglobulin reactivity due to polyclonal rheumatoid factors. *Cancer Res.*, **47**, 4520.

DESHPANDE, S.V., DENARDO, S.J., KUKIS, D.L. & 4 others (1990). Yttrium-90 labelled monoclonal antibody for therapy: labelling by a new macrocyclic bifunctional chelating agent. *J. Nucl. Med.*, **31**, 473.

EPENETOS, A.A., BRITTON, K.E., MATHER, S. & 8 others (1982). Targeting of Iodine-123-labelled tumour-associated monoclonal antibodies to ovarian, breast and gastrointestinal tumours. *Lancet*, **ii**: 999.

EPENETOS, A., COURTENAY-LUCK, N.S., HALNAN, K.E. & 17 others (1984). Antibody guided irradiation of malignant lesions: three cases illustrating a new method of treatment. *Lancet*, **i**, 1441.

EPENETOS, A.A., MUNRO, A.J., STEWART, S. & 14 others (1987). Antibody guided irradiation of advanced ovarian cancer with intraperitoneally administered radiolabelled monoclonal antibodies. *J. Clin. Oncol.*, **5**, 1890.

EPENETOS, A.A., SNOOK, D., HOOKER, G. & 5 others (1985). Indium-111 labelled monoclonal antibody to placental alkaline phosphatase in the detection of neoplasms of testis, ovary and cervix. *Lancet*, **ii**, 350.

EPENETOS, A.A., TRAVERS, P., GATTER, K.C., OLIVER, R.D.T., MASON, D.Y. & BOAMER, W.F. (1984). An immunohistological study of testicular germ cell tumours using two different monoclonal antibodies against placental alkaline phosphatase. *Br. J. Cancer*, **49**, 111.

HIRD, V., SNOOK, D.E., KOSMAS, C. & 6 others (1991). Intraperitoneal radioimmunotherapy with stable immunoconjugates of yttrium-90. *Advances in the Applications of Monoclonal Antibodies in Clinical Oncology*, Epenetos, A.A. (ed.), Chapman & Hall: UK, p. 267.

KOSMAS, C., SNOOK, D., GOODEN, C.S. & 4 others (1991). Development of humoral immune responses against a macrocyclic chelating agent (DOTA) in cancer patients receiving monoclonal antibodies for imaging and therapy (Submitted for publication).

MCCALL, M.J., DIRIL, H. & MEARES, C.F. (1990). Simplified method for conjugating macrocyclic bifunctional chelating agents to antibodies via 2-iminothiolane. *Bioconjugate Chem.*, **1**, 222.

MEARES, C.F., MOI, M.K., DIRIL, H. & 6 others (1990). Macrocyclic chelates or radiometals for diagnosis and therapy. *Br. J. Cancer*, **62** (Suppl X), 21.

MOI, M.K., MEARES, C.F. & DENARDO, S.J. (1988). The peptide way of macrocyclic bifunctional chelating agents. Synthesis of 2-(p-Nitrobenzyl) - 1,4,7,10 - tetracyclododecane - N,N',N'',N''',N'''' -tetraacetic acid, and study of its Yttrium (III) complex. *J. Am. Chem. Soc.*, **110**, 6266.

RIECHMANN, L., CLARK, M., WALDMANN, H. & WINTER, G. (1988). Reshaping human antibodies for therapy. *Nature*, **332**, 323.

SNOOK, D.E., ROWLINSON-BUSZA, G., MEARES, C. & EPENETOS, A.A. (1991). Preparation and biodistribution of indium-111 and yttrium-90 labelled immunoconjugates incorporating the macrocyclic chelating agent DOTA. In *Monoclonal Antibodies: Applications in Clinical Oncology*, 16. Epenetos, A.A. (ed.), Chapman & Hall: UK, pp. 157–166.

STEWART, J.S.W., HIRD, V., SNOOK, D. & 11 others (1990). Intraperitoneal Yttrium-90 labelled monoclonal antibody in ovarian cancer. *J. Clin. Oncol.*, **8**, 1941.

TRAVERS, P. & BODMER, W.F. (1984). Preparation and characterisation of monoclonal antibodies against placental alkaline phosphatase and other human trophoblast-associated determinants. *Int. J. Cancer*, **33**, 633.

VERHOEYEN, M., BRODERICK, L., EIDA, S. & BADLEY, A. (1991). Reshaping human antibodies for cancer diagnosis and therapy: grafting an anti-human PLAP specificity. *Monoclonal Antibodies: Applications in Clinical Oncology*, 5. Epenetos, A.A. (ed.), Chapman & Hall: UK, pp. 37–43.